# Editorial: Human movement coordination in healthy and pathological conditions: from neuromuscular and kinetic principles to muscle-tendon function

**DOI:** 10.3389/fspor.2025.1623815

**Published:** 2025-05-21

**Authors:** Maria-Elissavet Nikolaidou, Lida Mademli, Kiros Karamanidis, Adamantios Arampatzis

**Affiliations:** ^1^ Sports Biomechanics Laboratory, Faculty of Physical Education and Sport Science, School of Physical Education and Sport Science, National and Kapodistrian University of Athens, Athens, Greece; ^2^ Laboratory of Adapted Physical Education, Faculty of Physical Education and Sport Sciences, School of Physical Education and Sport Science (Serres), Aristotle University of Thessaloniki, Thessaloniki, Greece; ^3^ Department of Sport Science, Faculty of Mathematics and Natural Sciences, University of Koblenz, Koblenz, Germany; ^4^ School of Applied and Health Sciences, London South Bank University, London, United Kingdom; ^5^ Department of Training and Movement Sciences, Humboldt-Universität zu Berlin, Berlin, Germany; ^6^ Berlin School of Movement Science, Humboldt-Universität zu Berlin, Berlin, Germany

**Keywords:** human movement, kinematics, neuromuscular, muscle-tendon, coordination

**Editorial on the Research Topic**
Human movement coordination in healthy and pathological conditions: From neuromuscular and kinetic principles to muscle-tendon function

Effective and safe movement in complex environments relies on task-specific limb coordination with high temporal and spatial variability; it is also crucial for independent living. Regular exercise and repeated exposure trigger neural and musculoskeletal adaptations, leading to new or refined coordination patterns. Understanding these principles requires integrating knowledge from neuromuscular circuits to joint mechanics and muscle-tendon function. This Research Topic in Frontiers in Sports and Active Living focuses on current research exploring how human movement coordination in both healthy individuals and in those with pathological conditions is organized from a neuromuscular perspective to kinetic principles and to the level of muscle-tendon function.

In a comprehensive study, Fischer et al. investigated lumbo-pelvic posture and coordination, trunk dynamic stability, and movement variability in asymptomatic and chronic low back pain participants. Clear sex-specific differences in lumbo-pelvic coordination and posture measures were found, while pain intensity had only a small effect, indicating the limited utility in the clinical diagnosis and management of low back pain of these variables. Their findings of greater local trunk instability in the participants with chronic low back pain, which predict control errors in the regulation of trunk movement, could be considered as a useful diagnostic tool. Hakamata et al. used immersive virtual reality to manipulate the placement of the leading limb, examining whether controlling its position could facilitate the need to prioritize movement planning of the trailing limb during a virtual obstacle-crossing task. Their findings suggested a reduction in the collision rate of the trailing limb as a result of a safer motor strategy of obstacle avoidance by the leading limb, thus highlighting the trade-off between speed and accuracy, which older adults could be trained to effectively execute. Köhler et al. investigated the kinetics of the javelin throw and found that the energy generated during the acceleration phase significantly influences joint moments and energy transfer in the subsequent deceleration phase. Their study highlights the crucial role of the acceleration phase in optimizing energy flow and reducing injury risk while ensuring the athlete's better technical preparation. The work by Steingrebe et al. offers a comprehensive review and meta-analysis of hip osteoarthritis on lower limb joint angles in the three planes of motion during locomotor tasks of varying difficulty involving gait, stair walking, and turning. Overall, this work highlights the complex interplay of kinematic changes affecting both the ipsilateral and contralateral sides as well as the task-specific kinematic alterations during different phases of locomotion.

Biomechanical and sensorimotor orthotic interventions have distinct roles depending on the action mechanisms of the joint kinematics of the lower extremities. The study by Simon et al. aimed to analyze the effects of biomechanical and sensorimotor foot orthoses on gait kinetics in patients with patellofemoral pain through a randomized controlled trial. Their results revealed that sensorimotor foot orthoses induced distinct kinematic adaptations in lower extremity motion, potentially reducing patellofemoral pain during walking. In their contribution, Pfile et al. investigated the effect of the technique of anterior cruciate ligament reconstruction with a quadriceps tendon autograft during gait in patients with unilateral injury almost one-year post-surgery. Their findings showed significant biomechanical alterations and gait patterns associated with quadricep avoidance and diminished proximal forces during specific phases of gait. The role of the “upper body strategy” as a possible compensatory mechanism to the impaired dynamic balance performance following exercise-induced lower limb muscle fatigue was examined in healthy youth by Borgmann et al., where findings showed that free arm movement did not moderate the impact of neuromuscular fatigue on dynamic balance performance.

Mademli et al. investigated the diversity and flexibility of activation patterns within the synergistic triceps surae and quadriceps muscles during a visually guided postural task in stable and unstable conditions. Although the similarity of activation patterns of the synergistic muscles decreased during the unstable condition, the lower values within the triceps surae muscles in both examined conditions indicate increased flexibility and diversity of neuromuscular control to meet specific joint stabilization challenges. Liang et al. focused on the chronic adaptation of the biomechanical properties of the Achilles tendon in long-term post-stroke patients. They reported region-specific degeneration in tendon thickness and reduced collagen fiber organization in the paretic limb, increasing the risk for the initiation of tendinopathy and, thus, supporting more targeted rehabilitation strategies to cope with the impaired lower extremity function typically characterizing this population. Further, Magris et al. assessed the intrinsic and morphological properties of vastus lateralis muscle in patients with Parkinson's disease and found altered force generation capacity of the muscle during contraction, whereas muscle mechanical power emerged as a potential parameter useful for clinical evaluation between the less and the more affected side. Kim et al. synthesized the current evidence on the effect of sensorimotor training on pain and functional outcomes in the Achilles tendon where, despite the low number of included studies, potential positive effects of sensorimotor training in conjunction to high loading strategies were shown with regards to pain, strength, and short-term performance outcomes. Changes in hormonal concentrations during the menstrual circle have been suggested as a possible causal factor for the altered mechanical properties in tendons amounting to a higher injury rate in females. In their contribution, Saito et al. found that muscle as well as tendon stiffness in the anterior and lower posterior thigh regions were unaffected by menstruation, although a positive correlation only in the anterior thigh region with the early luteal phases of menstruation was found. Finally, the systematic treatment of eccentric muscle function as a training modality in sports and rehabilitation due to well-documented acute and prolonged adaptations has been sufficiently investigated. Vila-Chã et al. focused on the less explored neuromuscular and temporal alterations of eccentric exercise and their motor-task specificity and showed that isotonic eccentric exercise induced different responses depending on the investigated neuromuscular functional outputs and motor tasks.

The article collection presents findings that contribute to our understanding of the principles of human movement control and coordination in both healthy individuals and those with pathological conditions. Collectively, the results point to significant advances in targeted exercise or rehabilitation strategies. Yet, due to the complex interaction between human motor control at the neuromuscular, joint-limb, and muscle-tendon levels and their resulting modulation and/or adaptation to environmental demands, many issues require further investigation. Some studies suggest that more sensitive and clinically useful measures are needed to better clarify adaptations in temporal and spatial motor responses as well as in the intrinsic properties of the neuromuscular system between patients and asymptomatic controls in pathologies like chronic low back pain, Parkinson's disease, or stroke. Likewise, it is necessary that these altered functional coordination patterns be investigated in experimental designs able to manipulate task complexity, populations with different functional levels, and populations at various stages of recovery. We would like to thank all contributors for their participation to this Research Topic and look forward to joining our efforts in human movement coordination research.

